# Health-related quality of life of patients after ischaemic stroke treated in a provincial hospital in Poland

**DOI:** 10.1080/20016689.2020.1775933

**Published:** 2020-06-17

**Authors:** Szymon Jarosławski, Bożena Jarosławska, Barbara Błaszczyk, Pascal Auqier, Mondher Toumi

**Affiliations:** a Public Health Department – Research Unit EA 3279, Aix-Marseille University, Marseille, France; b Dr. T. Chałubiński District Hospital, Zakopane, Poland; c Private Neurology Practice, Kielce, Poland

**Keywords:** Stroke, quality of life, depression, anxiety, cognitive function

## Abstract

**Background:**

Ischaemic stroke (IS) is a major cause of death and disability and affects the quality of life of patients. Previous studies focused on urban populations.

**Objective:**

To evaluate the health-related quality of life (QoL) of patients with history of IS and living in a rural area in Poland.

**Patients:**

Rural population of 172 patients discharged from a district hospital in Zakopane, Poland with a diagnosis of IS in the period from 01.01.2005 to 31.10.2006.

**Intervention:**

QoL was evaluated using the European Quality of Life Scale-5 Dimensions EQ-5D-3 L (EQ-5D) and the Short Form Health Survey – 12 version 2 (SF-12).

**Results:**

In the EQ-5D survey, 57.3% of patients had only some problems with mobility, 40.3% with usual activities, 63.2% with pain/discomfort, 59% with anxiety/depression, and 32.2% with self-care. In the SF-12 survey, both summary components (physical and psychological) were reduced compared to the population norm.

**Conclusion:**

The quality of life in IS survivors is clearly reduced in the majority of domains assessed by the EQ-5D and SF-12 questionnaires. The most important factors affecting QoL were the functional state, depression and anxiety. A significant difference as compared to to urban and mixed populations was observed for a reduced SF-12 mental health component and for the EQ-5D visual analogue scale. We found no effect of gender, age or cognitive disorders on the outcomes of SF-12.

## Introduction

Stroke is the second leading cause of death after ischemic heart disease and is characterized by high levels of disability among survivors [1,2]. During the first year after stroke, 25% of patients die and half of the survivors have a disability [3].

Quality of life was defined by the World Health Organization (WHO) as ‘An individual’s perception of their position in life in the context of the culture and value systems in which they live and in relation to their goals, expectations, standards and concerns. It is a broad ranging concept affected in a complex way by the persons’ physical health, psychological state, level of independence, social relationships, and their relationship to salient features of their environment’ [4].

In medical sciences, the term health-related quality of life (QoL) was introduced by Schipper in 1990 [5]. Numerous validated tools are available for assessing QoL. The most commonly used generic tools are the Medical Outcomes Short Form Health Survey (SF-36) [6], and its abbreviated SF-12 version [7], the Sickness Impact Profile (SIP) [8] and the European Quality of Life Scale-5 Dimensions (EQ-5D) [9]. Stroke-specific tools have also been created, such as the Stroke-Adapted Sickness Impact Profile (SA-SIP30) [8] or the Stroke Impact Scale (SIS) [10,11]. However, there is significantly less research experience with these scales, and their Polish adaptations were not available at the time of the current study.

Experiencing a stroke significantly worsens quality of life of patients [12]. The 2001 Expert Report of the Polish National Program of Prophylaxis and Treatment of Brain Stroke recommends assessing quality of life during post-stroke rehabilitation [13]. However, medical personnel’s knowledge of QoL scales can be limited. In 2003, a survey of knowledge of QoL tools among 1572 doctors and physiotherapists in the UK showed that 72% of the respondents understood this term as happiness, 91% preferred asking the patients directly about their quality of life, and only 40% recognized the need to use evaluation tools [14]. While this awareness might have improved ever since this data is relevant to the period of the current study.

Authors of numerous assessments of patients after stroke agree that QoL of these patients is clearly declining, notably in most of the functioning and mental well-being domains [15–19]. However, they are not in agreement as to the importance of factors determining QoL after stroke. The authors report parameters that can influence QoL, such as age, sex, functional status including the degree of disability, the presence of depression, social status, and social support [15,17–23]. Evidence is available that motor and cognitive impairments after stroke are correlated [24]. The presence of cognitive impairment causes greater helplessness than the motor deficit and can reduce patients QoL [13,25].

Research on QoL in stroke survivors in Poland has been typically carried out in populations of large-city dwellers or in mixed rural-urban populations, but with an over-sampling of the urban population [18,19,26,27]. However, epidemiological studies show that city and rural populations differ not only in terms of demographics but also in many socio-economic factors, such as income, household size, social ties, etc [28,29]. The aim of this study was to assess the QoL of stroke patients in the rural population in Southern Poland and its correlation with the consequences of stroke, such as paresis, depression, cognitive impairment and others.

## Methodology

Based on the medical documentation of the Dr. T. Chałubiński District Hospital in Zakopane, a register was set up of all cases of patients discharged from the hospital in the period from 1/1/2005 to 31/10/2006 with a diagnosis of IS (I63 according to ICD 10), who were over 18 years old, and resided in the Tatra or Nowy Targ districts. The study population was covered by a universal health insurance provided by the National Health Fund (NFZ). The study was exempted from an ethics committee approval because the scope of medical examination of patients did not differ from what is recommended in a regular medical follow up of stroke survivors.

In addition to demographic data, risk factors prior to the occurrence of the stroke, the clinical data regarding the stroke, the degree of disability on the modified Rankin scale [30] and the recommended secondary stroke prevention measures [13] were extracted from medical records.

In total, 256 patients were discharged from the hospital with IS diagnosis. Based on information from the official population records it was established that during 6–12 months from hospital discharge 31 patients died. The remaining 225 patients were sent a personal invitation to participate in the study via mail. One hundred and seventy-two people gave consent to participate in the study. Patients came to the hospital’s outpatient neurological clinic in Zakopane at a time convenient to them or were examined by a neurologist (BJ) in their place of residence. During the assessment, which took place 6–18 months after stroke, demographic data were collected (age, education level, current employment, reliance on assistance by others). In addition, a general physical and a neurological interview and examination were carried out, as well as assessments of symptoms of anxiety, depression and QoL.

In the general physical examination, the following parameters were measured: blood pressure (twice, at the beginning and at the end of the examination), heart rate, weight, height and waist circumference. The Body Mass Index (BMI) was calculated based on the weight and height measurements. Abdominal type obesity was defined as waist circumference above 102 cm in men and above 88 cm in women.

In the neurological assessment, the presence and severity of limb and face paresis and speech disorders were evaluated. The functional state of the patients was assessed using the 100-point Barthel Index (BI) with five functional disability scores proposed by Jørgensen et al. [21]. The presence of symptoms of anxiety and depression was evaluated using the 14-point Hospital Anxiety and Depression Scale (Hospital Anxiety and Depression Scale, HADS), which has been validated in stroke patients [31,32]. Cognitive impairment was assessed by the Mini Mental State Examination (MMSE) test [33]. The result of 24 points and below was taken as an indicator of dementia.

QoL was evaluated using the Polish versions the EQ-5D-3 L questionnaire [34] and the SF-12 scale version 2 [35]. The EQ-5D questionnaire consists of two parts. The first part assesses quality of life in five dimensions: mobility, self-care, usual activities, pain/discomfort and anxiety/depression. The EQ-5D index was calculated based on the widely accepted standards for the UK and the Polish population standards [34]. In the second part (EQ-VAS), the patient is asked to mark their own assessment of the current state of health on a visual-analogue scale ranging from 0 to 100, where the minimum means the worst and the maximum – the best conceivable health.

The SF-12 scale evaluates quality of life in two dimensions: mental and physical. Each dimension consists of four subscales, each with a maximum of 50 points. Physical Health Scores – (PHS) contains the following subscales: General Health (GH), Physical Functioning (PF), Role Physical (RP), and Body Pain (BP). Mental Health Scores (MHS) contains 4 subscales: Vitality (VT), Social Functioning (SF), Role Emotional (RE), and Mental Health (MH). The Physical Composite Scale (PCS) and the Mental Health Composite Scale (MCS), are averages of subscales of the corresponding categories.

Rating on the SF-12 scale it is based on an external standard. It has been shown that the USA population standard is not significantly different from the ones calculated for populations of nine European countries [36]. Therefore, we adopted the USA standard published in 1998. The SF-12 can be used in all patients regardless of the severity of stroke [37].

Means, standard deviations and percentages were used to describe the questionnaire scores. We used the Student’s t test to analyse the relationships when the independent variables were dichotomous. In case of numerical variables, we used the Pearson’s linear correlation coefficients. In order to identify the independent prediction factors, a multiple regression method was applied. We assumed 0.05 as the level of statistical significance. SF-12 data was analysed using the dedicated software supplied by the survey owner (SF-12v2 Health Survey, Scoring software 2.0, QualityMetric Incorporated). Patients’ knowledge about the principles of secondary stroke prevention and compliance were assessed using a questionnaire (Table 1).

**Table 1.** Knowledge of the principles of secondary prevention of stroke and compliance with its recommendations.

Area of knowledge of and compliance with the recommendations received at hospital discharge	n	%
Knowing your blood pressure	120	69.8
Knowledge of the range of a normal blood pressure	18	10.5
Measurement of blood pressure at home	118	68.6
Knowledge of weight	135	78.5
Knowledge of appropriate weight range	102	59.3
Knowledge of your glucose levels	42	24.4
Knowledge of normal glucose levels range	38	22.1
Knowing your cholesterol level	28	16.3
Knowledge of normal cholesterol level range	21	12.2
Quit smoking	11	32.4 (% of smokers)
Limited smoking	12	69.8
Lost weight	38	22.1
Increased physical activity	38	22.1
Changed diet	50	29.1
Prevention of secondary stroke at the time of assessment:		
Only aspirin	132	76.7
Aspirin and other anti-platelet medication	15	8.7
Ticlopidine	1	0.6
Anticoagulants	37	88.1 (% of patients with AF)
The lack of pharmaco-prevention	11	6.4
Statin drugs	77	44.8
Endarterectomy	1	0.6
Angioplasty	3	1.7
Rehabilitation	96	55.8
Speech therapy	7	13.2 (% of patients with aphasia)

AF: atrial fibrillation

## Results

### Patients socio-demographic data

All 172 patients (n = 90 female) who consented to the study were included in the analysis, which constituted 76.4% of the patients who survived more than 6 months after the stroke. Only one patient was a resident of a residential care home. Age ranged from 34 to 95 years (the average age at the time of stroke occurrence was 70.5 years); women were on average 4 years older than men. Among the respondents, 69 (40.1%) were 75 years of age or older, 113 (65.7%) patients had no higher than primary education, 42 (24.4%) no higher than secondary education and only 4 (2.3%) had higher education. One hundred and sixty patients (93%) were retired or perceiving a pension when the stroke occurred.

### Patients health status at hospitalization due to stroke

During hospitalization, hypertension was diagnosed in 127 (73.8%) patients, atrial fibrillation in 42 (24.4%), and diabetes in 37 (21.5%). On discharge from the hospital, 122 (70.9%) patients had hemiparesis, 53 (30.8%) had aphasia of various degrees, and 14 (8.1%) dysarthria. Considerable disability (score 3 and higher on the Rankin scale) was present in 45 (26.2%) patients; small disability (score 2 on the Rankin scale) in 39 (22.7%), insignificant disability (score 1 on the Rankin scale) in 80 (46.5%). Complete functional recovery was reported in 8 (4.6%) patients. As part of prevention of secondary stroke after discharge, 38 of 42 (90.5%) patients with diagnosed atrial fibrillation were recommended anticoagulant therapy. The remaining 134 patients without atrial fibrillation were recommended antiplatelet treatment, mainly aspirin.

### Patients assessment at 6–18 months from stroke

At 6–18 months from the stroke occurrence, 16 (9.3%) patients experienced a subsequent IS during, of which 14 (8.1%) were hospitalized as a result of the stroke, and 2 (1.2%) were treated in an outpatient setting. A transient ischemic attack (TIA) occurred in 18 patients (10,5%), of which 14 (8.1%) were treated in a hospital.

Hemiparesis persisted in 92 (53.5%) patients, speech disorders in 38 (22.1%). Seven out of 52 patients with aphasia at hospital discharge (13.2%) underwent post-stroke speech therapy. Total return to health from before stroke (100 points on the BI) was found in 58 (33.7%) patients, mild disability (75–95 points) in 80 (46.5%), moderate disability (50–70 points) in 14 (8.1%), severe disability (25–45 points) in 11 (6.4%), and very severe disability (0–20 points), in 9 (5.2%) patients. Ninety-six (55.8%) patients underwent post-stroke rehabilitation.

Increased systolic blood pressure was found in 72 (41.9%) and diastolic blood pressure in 34 (19.8%) patients. Due to significant narrowing of the internal carotid artery, endarterectomy was performed in one patient and angioplasty with stenting in three more patients, both without complications. The study found obesity (BMI> 30) in 50 (29.1%) patients. Abdominal type obesity was found in 16 (19.5%) male and 51 (56.7%) female patients.

Patients’ knowledge about the principles of secondary stroke prevention and compliance are presented in Table 1.

On the basis of the HADS scale, symptoms of anxiety were present in 67 (39.6%), and of depression in 55 (32.5%) patients. Only one patient was receiving pharmacological treatment for depression. Dementia (less than 24 points on the MMSE scale) was found in 62 (38.3%) patients.

**Figure 1.**
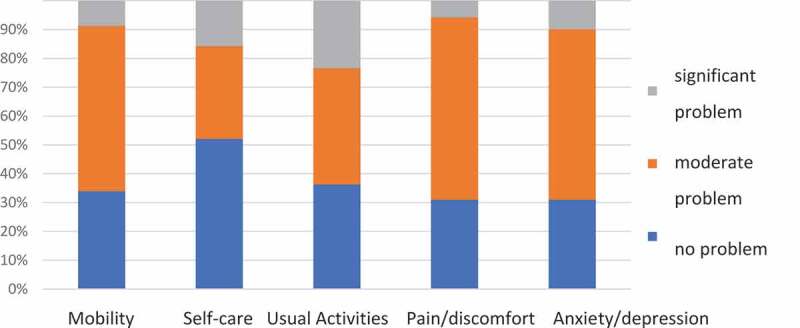
Results of the EQ-5D survey. Sample size n = 171.

One out of the 172 patients (0.6%) was excluded from the EQ-5D study due to significant aphasia. The results of the EQ-5D questionnaire are shown in Figure 1. Nine-eight (57.3%) patients had only some problems with mobility, 69 (40.3%) with usual activities, 108 (63.2%) with pain/discomfort, 101 (59%) with anxiety/depression, and 55 (32.2%) had problems with self-care. Significant problems in these domains were reported by a smaller percentage of patients. In particular, 40 (23.4%) patients had significant problems in the usual activities domain and 27 (15.8%) in the self-care domain. The EQ-5D index calculated based on the UK standard was 0.51 and based on the Polish standard – 0.68.

**Figure 2.**
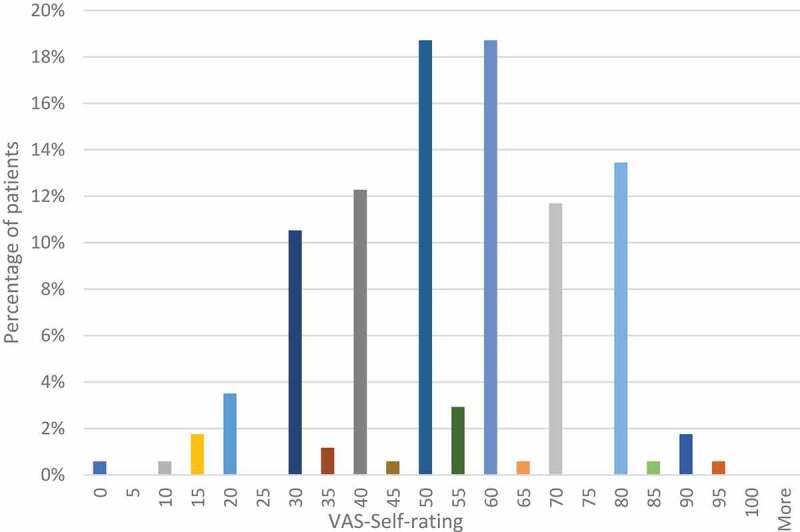
EQ-VAS results. Sample size n = 171.

The results of the assessment with the visual-analogue scale (EQ- VAS) are shown in Figure 2. The patients assessed their current condition most often in the range of numbers from 50 to 70 (average: 54.4). Only a small percentage of patients rated their health in values below 20 (2.9%) or above 80 (1.7%).

Factors affecting the results of the EQ-5D assessment (except VAS) are presented in Table 2. In the univariate analysis, depression and speech problems were predictors that affected all dimensions of the questionnaire. Most dimensions were also affected by other factors, such as the presence of paresis or subsequent stroke. For example, in the univariate analysis, all dependent variables except education level and previous stroke had an impact on mobility. Gender did not predict any of the EQ-5D dimension scores.

**Table 2.** Summary of factors affecting the results of the evaluation with questionnaires EQ-5 D and SF-12 using univariate and multivariate analysis.

	Age	Education	Previous stroke	Rankin scale rating	The presence of paresis	Speech disorders	Barthel scale assessment	Anxiety	Depression	Subsequent stroke	Dementia
EQ mobility	x			x	x	x	X *	x	X *	x	x
EQ self-care	x		x	x	X *	x	X *		X *	x	x
EQ usual activities	x			x	X *	x	X *		X *		X *
EQ pain/discomfort	X *				x	x	x	X *	x		x
EQ anxiety/depression						X *	x	*	X *		x
EQ VAS						x	x	X *	X *	x	x
SF PCS	x		X *	x	X *	x	X *		X *	x	x
SF MCS							x	X *	X *		x
SF PF	x		x	x	X *	X *	X *	x	X *	x	x
SF RP	x		x	x	x		X *		X *	x	x
SF BP		X *					x	X *	X *		x
SF GH			X *			x	x	X *	X *		x
SF VT			x	x	x		x	X *	X *	x	x
SF SF							x	X *	X *	x	
SF RE							X *	X *	X *		x
SF MH	x						X *	X *	X *		

* – confirmed with regression test

x- univariate analysis

X- multivariate analysis

EQ: EuroQol-5D, SF: Short Form-12, BP: Body Pain, GH: General Health, MCS: Mental Health Composite Scale, MH: Mental Health, PCS: Physical Composite Scale, PF: Physical Functioning, RE: Role Emotional, RP: Role Physical, SF: Social Functioning, VT: Vitality

The complete numeric data is available as Supplementary Material 1.

In the multivariate analysis using the multiple regression coefficient, the scores on individual EQ-5D domains were predicted by the presence of paresis, dementia, depression and anxiety (Table 2; Supplementary Material 1).

Due to significant aphasia, three patients were excluded from the SF-12 assessment due to the higher complexity of SF-12 as compared to EQ-5D. In the resulting sample of 169 patients, 91 (54%) were women and the age ranged from 35 to 95 years with a mean of 71 years. The complete results of the SF-12 survey are shown in Supplementary Material 2. The PCS mean score was 34.4 and the MCS was 43.9 (Table 3).

**Table 3.** **Results of the SF-12 survey**. Sample size n = 169.

		Physical Health Scores				Mental Health Scores			
PCS	MCS	PF	RP	BP	GH	VT	SF	RE	MH
34.37	43.90	35.88	34.03	44.36	30.73	34.59	42.77	42.49	44.02

Values are mean scores. BP: Body Pain, GH: General Health, MCS: Mental Health Composite Scale, MH: Mental Health, PCS: Physical Composite Scale, PF: Physical Functioning, RE: Role Emotional, RP: Role Physical, SF: Social Functioning, VT: Vitality. Further details can be consulted in Supplementary Material 2.

In relation to the population standard, lower scores were obtained for the PCS subscales GH, RP and PF. In the PCS category, only 15% of patients were within the adopted norm, and 85% of respondents obtained inferior scores (Supplementary Material 2: Figure 1). Slightly higher scores were reported in the MCS dimension, where 50% of respondents were below the norm, 22% within the norm and 28% had scores above the norm (Supplementary Material 2: Figure 1).

People aged 45–64 years gave the lowest ratings for their physical health and younger patients, aged 35–44 years old, for their mental health. The highest mental health scores were reported in the oldest group of patients, over 75 years old. However, patients’ age or gender did not impact the result of the SF-12 survey significantly (Supplementary Material 2: Figures 2 and 3).

As for the EQ-5D scores, the results of individual dimensions and subscales of SF-12 were influenced by various variables in the univariate analysis (Supplementary Material 1). The PCS was influenced by all analysed variables apart from education level and anxiety, and the MCS was influenced only by some variables, i.e. degree of disability, anxiety, presence of depression and dementia. The relationship between reduced scores on the perceived pain (BP) domain and low education level was confirmed in the regression test (Supplementary Material 1).

In the multi-variate analysis, physical health dimension scores were affected by subsequent stroke, presence of paresis, speech disorder, anxiety and depression (Supplementary Material 1). The mental health components were affected by the degree of disability and the presence of anxiety and depression (Supplementary Material 1).

## Discussion

The quality of life in IS survivors is clearly reduced in the majority of domains assessed by the EQ-5D and SF-12 questionnaires. These findings are consistent with results of many previous researchers assessing different populations, mainly patients from Western European and North American countries using different scales, as discussed below. The same QoL tools as in the presented work had been used by of several authors [16,35,38–40]. We discuss also SF-36 studies because it has been shown that both SF scales can be mutually reproduced [39].

However, none of these studies used the Polish versions of the assessment tools. The Polish studies on the QoL in stroke survivors used the Sickness Impact Profile (SIP) [17–19,27]. Further, one study used the Ferrans and Powers Standardised Questionnaire Index of the Quality of Life [26,41]. These studies showed that after 6 months of stroke, most of the quality of life domains were reduced [18,19,41] and that functional disability and depression were independent predictors of the QoL [17]. However, the study populations were patients admitted to hospitals in large cities and were predominantly urban dwellers [19,26,27].

In the present study, patients showed reduced SF-12 summary scores and subscales, in particular: General health (GH), Role Physical (RP) and Physical Functioning (PF). Similar results were obtained in other stroke studies [15,16,38,39,42–44]. In particular, Gray et al. [38] examined 1268 patients 6 months after IS and obtained a PCS score of 36.4 (versus 34.4 in our study). A similar result of 35.6 was shown in an American study on 39 680 patients with all types of stroke [16]. An exception was a study in Germany that showed a PCS only slightly inferior to the population standard [45].

However, our results differ from previous work in terms of the mental health component summary (MCS). Our work reported a mean score of 43.9 which is reduced compared to the population norm of 53 points. Most researchers obtained a result close to the population standard [16,35,38] and others obtained results even above the standard [40]. Only one study assessing 314 patients in Germany after various kinds of stroke, including 69% of IS patients, showed a reduced MCS at 12 months from stroke [45].

In terms of the EQ-5D assessment, the scores for patients’ mobility, self-care and everyday activities are similar to a previous study [39]. Further, the EQ-5D index (global summary score) was 0.68 in our study and was comparable to 0.69 [16] or 0.62 [39,42] in previous work. Clearly, these results are inferior to the Polish population norms established at 0.82 in the 65–74 years old age group and at 0.73 in the 75 years old and above age group and also to the US population norms (0.82 and 0.76 respectively) [46,47].

Nevertheless, the EQ-VAS mean of 54.4 in our study is strikingly below the 70.0 [39], 60.0 [48] and 61.6 [16] means reported by other researchers. However, the Polish population norm for the EQ-VAS was established at 62.8 in the 65–74 age group and at 54 in the 75 years old and above age group [46]. Therefore, while still somewhat inferior to the Polish population norm, our relatively low EQ-VAS results could be at least partially explained by the generally poorer self-perceived health status of the Polish elders, as compared to those living in the US where the referenced studies had been carried out. Indeed, the US EQ-VAS population norms in these age groups were much higher, at 75 and 68 respectively [47].

It is also possible that, in our study population, the EQ-VAS captured certain aspects of patients self-perceived reduction in QoL that was not fully reflected in any of the five domains of the EQ-5D questionnaire [49]. Possibly, since the SF-12 MCS in our population was lower than in other studies, this reduction could correspond to a worsening of the mental health after stroke that was not fully captured by the EQ-5D domains. Interestingly, the EQ-VAS component has been shown to have a weaker correlation with BI and the modified Rankin scale than the EQ-5D domains [50]. Indeed, in our study the EQ VAS was significantly affected by both depression and anxiety symptoms, but not by any other variables (Table 2). The utility of the EQ-VAS component for assessing the mental health dimensions of QoL in stroke patients warrants further research.

Further, the incidence of depressive disorders in stroke patients was estimated in a meta-analysis of 96 publications from 1997–2002 at 33% (29% −36%), depending on the time interval from the stroke [51]. This is similar to the incidence of 32.5% reported in the current study. However, a previous Polish study at an urban university hospital showed depressive symptoms in a larger proportion of stroke patients – 47.8% [19].

The results of our study confirm the importance of mental well-being in QoL in IS patients, as depression impacted almost all surveyed QoL domains. The dependence of QoL on depression was confirmed by the regression analysis in most cases, except for the EQ pain/discomfort domain.

Indeed, depression is mentioned as the most important predictor of QoL by many authors [20,29,37,52,53]. As shown by Kauhanen et al. [15] depression impacted negatively the SF-36 VT and RP domains, whereas and in a different study, all SF-36 domain scores were lower if depression was present, in the same way as in our study [44]. However, in our sample, only one among 55 patients presenting depressive symptoms received pharmacotherapy for depression, which shows that the diagnosis and/or treatment of depression in IS survivors is underestimated by physicians who follow stroke patients in the study setting. While non-pharmacological treatments for post-stroke depression have also been reported by authors [54], these are not routinely available in the public health care system in rural Poland.

Further, many publications have highlighted the significant influence of neurological deficit (paresis, aphasia) on quality of life [29,48,55]. Some authors consider that a serious neurological deficit predisposes patients to depression, which in turn affects QoL [19].

Stroke survivors have twice the risk of developing dementia than subjects from a control population group [56]. The incidence of dementia in urban population stroke studies was estimated at 30% [57]. In the rural population studied here, dementia was found in 37.9% of patients. Since having more education years is a known protector against dementia [58] the higher prevalence of dementia in the current study group could be explained by the fact that that 65.7% patients had no higher than primary education, as compared to only 31% in the general population aged 15 years old and older in Poland [59]. However, the cognitive impairment generally did not impact the QoL of patients after stroke: there was a significant relationship only with the EQ5D – usual activities domain. This largely confirms the observations of others authors who have shown a lack of dependence of QoL on the cognitive dysfunction [15,29,48]. Also, in line with previous studies [44,57] the level of education was not a determinant of QoL in our study.

Finally, like in many previous studies, we did not find any relationship between the quality of life and gender [15,20,40,44,48].

The authors are aware of limitations of this study. For example, patients were evaluated in various periods from the IS incident. Due to the lack of similarly designed studies, we have compared our results to those that were the closest to us our setting, including research in other periods after stroke and in all types of strokes. Further, we did not assess the role of social support in patients’ QoL. Also, no stroke-specific QoL instrument was used. Finally, the size of the sample of patients was relatively small and the presented data is over a decade old.

## Conclusions

The presented work shows the importance of the assessment of QoL in patients after stroke, alongside the assessment of treatment and rehabilitation effects. Importantly, the rural-dwelling patients in our study gave worse ratings for the mental health scores of SF-12 and on the EQ-VAS than predominantly urban populations in previous work. Interestingly, the prevalence of depression among rural stroke patients was smaller as compared to previous data from an urban university hospital setting in Poland. However, the diagnosis and/or treatment of depression in our study setting was largely neglected. Further, while the prevalence of dementia in the rural setting was higher than in previous studies in urban and mixed populations, the presence of dementia did not affect patients’ QoL significantly.

## Supplementary Material

Supplemental Material

## Supplementary material

The supplemental data for this article can be accessed here.
