# Supplementary material for: Health-related quality of life of patients after ischaemic stroke treated in a provincial hospital in Poland
Source: J Mark Access Health Policy. 2020 Jun 17;8(1):1775933. doi: 10.1080/20016689.2020.1775933 (PMC7482738; doi:10.1080/20016689.2020.1775933)

**Supplementary Material 1**

**Factors predicting quality of life after stroke – univariate analysis**

BP: Body Pain, GH: General Health, MCS: Mental Health Composite Scale, MH: Mental Health, PCS: Physical Composite Scale, PF: Physical Functioning, RE: Role Emotional, RP: Role Physical, SF: Social Functioning, VT: Vitality

red colour values: *p* < .05

blue colour values: *p* < .01

Rankin scale rating on hospital discharge and quality of life domains

| Dependent variables | Mean | | Standard Deviation | | N | | t | df | p |
| --- | --- | --- | --- | --- | --- | --- | --- | --- | --- |
|  | 0-2 | 3-5 | 0-2 | 3-5 | 0-2 | 3-5 |  |  |  |
| EQ mobility | **1.59** | **2.20** | **0.54** | **0.55** | **127** | **44** | **-6.46** | **169** | **0.000** |
| EQ self-care | **1.41** | **2.30** | **0.60** | **0.73** | **127** | **44** | **-7.99** | **169** | **0.000** |
| EQ usual activities | **1.67** | **2.45** | **0.68** | **0.70** | **127** | **44** | **-6.56** | **169** | **0.000** |
| **EQ pain/discomfort** | **1.70** | **1.89** | **0.55** | **0.54** | **127** | **44** | **-1.93** | **169** | **0.055** |
| EQ anxiety/depression | 1.76 | 1.86 | 0.64 | 0.51 | 127 | 44 | -0.94 | 169 | 0.348 |
| EQ VAS | 55.02 | 50.68 | 17.60 | 22.14 | 127 | 44 | 1.32 | 169 | 0.190 |
| **PCS** | **36.16** | **28.98** | **9.08** | **8.35** | **127** | **42** | **4.53** | **167** | **0.000** |
| MCS | 43.83 | 44.09 | 13.16 | 11.85 | 127 | 42 | -0.11 | 167 | 0.910 |
| **PF** | **47.24** | **18.45** | **29.59** | **26.51** | **127** | **42** | **5.60** | **167** | **0.000** |
| **RP** | **43.41** | **18.45** | **29.20** | **25.93** | **127** | **42** | **4.93** | **167** | **0.000** |
| BP | 68.70 | 65.48 | 25.78 | 29.71 | 127 | 42 | 0.68 | 167 | 0.500 |
| GH | 27.64 | 27.14 | 22.95 | 25.64 | 127 | 42 | 0.12 | 167 | 0.906 |
| **VT** | **19.29** | **11.31** | **23.19** | **16.75** | **127** | **42** | **2.06** | **167** | **0.041** |
| SF | 67.52 | 60.71 | 36.64 | 39.10 | 127 | 42 | 1.03 | 167 | 0.306 |
| **RE** | **70.08** | **59.23** | **30.01** | **36.74** | **127** | **42** | **1.92** | **167** | **0.057** |
| MH | 57.58 | 58.93 | 24.51 | 20.53 | 127 | 42 | -0.32 | 167 | 0.748 |

BP: Body Pain, GH: General Health, MCS: Mental Health Composite Scale, MH: Mental Health, PCS: Physical Composite Scale, PF: Physical Functioning, RE: Role Emotional, RP: Role Physical, SF: Social Functioning, VT: Vitality

red colour values: *p* < .05

blue colour values: *p* < .01

Education and quality of life domains

| Dependent variables | Mean | | Standard Deviation | | N | | t | df | p |
| --- | --- | --- | --- | --- | --- | --- | --- | --- | --- |
|  | P | I | P | I | P | I |  |  |  |
| EQ mobility | 1.79 | 1.67 | 0.56 | 0.69 | 113 | 58 | 1.18 | 169 | 0.239 |
| EQ self-care | 1.67 | 1.57 | 0.71 | 0.80 | 113 | 58 | 0.86 | 169 | 0.389 |
| EQ usual activities | 1.93 | 1.76 | 0.73 | 0.82 | 113 | 58 | 1.39 | 169 | 0.167 |
| EQ pain/discomfort | 1.78 | 1.69 | 0.55 | 0.57 | 113 | 58 | 1.00 | 169 | 0.321 |
| EQ anxiety/depression | 1.80 | 1.78 | 0.61 | 0.59 | 113 | 58 | 0.21 | 169 | 0.834 |
| EQ VAS | 53.79 | 54.14 | 18.54 | 19.74 | 113 | 58 | -0.11 | 169 | 0.909 |
| PCS | 33.50 | 36.09 | 9.24 | 9.57 | 112 | 57 | -1.71 | 167 | 0.090 |
| MCS | 44.22 | 43.26 | 12.57 | 13.37 | 112 | 57 | 0.46 | 167 | 0.648 |
| PF | 38.84 | 42.54 | 30.36 | 33.40 | 112 | 57 | -0.72 | 167 | 0.470 |
| RP | 36.16 | 39.25 | 30.40 | 30.38 | 112 | 57 | -0.63 | 167 | 0.532 |
| **BP** | **64.96** | **73.68** | **28.76** | **21.35** | **112** | **57** | **-2.02** | **167** | **0.045** |
| GH | 26.92 | 28.68 | 24.31 | 22.19 | 112 | 57 | -0.46 | 167 | 0.647 |
| VT | 15.63 | 20.61 | 20.72 | 24.15 | 112 | 57 | -1.40 | 167 | 0.164 |
| SF | 65.85 | 65.79 | 37.52 | 37.09 | 112 | 57 | 0.01 | 167 | 0.992 |
| RE | 68.19 | 65.79 | 33.60 | 28.98 | 112 | 57 | 0.46 | 167 | 0.646 |
| MH | 58.15 | 57.46 | 23.17 | 24.42 | 112 | 57 | 0.18 | 167 | 0.857 |

P: primary education only

I: higher than primary education

BP: Body Pain, GH: General Health, MCS: Mental Health Composite Scale, MH: Mental Health, PCS: Physical Composite Scale, PF: Physical Functioning, RE: Role Emotional, RP: Role Physical, SF: Social Functioning, VT: Vitality

red colour values: *p* < .05

blue colour values: *p* < .01

Speech disorder and quality of life domains

| Dependent variables | Mean | | Standard Deviation | | N | | t | df | p |
| --- | --- | --- | --- | --- | --- | --- | --- | --- | --- |
|  | yes | no | yes | no | yes | no |  |  |  |
| **EQ mobility** | **2.05** | **1.66** | **0.72** | **0.54** | **39** | **132** | **3.69** | **169** | **0.000** |
| **EQ self-care** | **2.08** | **1.51** | **0.81** | **0.67** | **39** | **132** | **4.44** | **169** | **0.000** |
| **EQ usual activities** | **2.26** | **1.76** | **0.75** | **0.73** | **39** | **132** | **3.72** | **169** | **0.000** |
| **EQ pain/discomfort** | **1.95** | **1.69** | **0.46** | **0.57** | **39** | **132** | **2.61** | **169** | **0.010** |
| **EQ anxiety/depression** | **2.05** | **1.71** | **0.51** | **0.61** | **39** | **132** | **3.15** | **169** | **0.002** |
| **EQ VAS** | **46.92** | **55.97** | **21.78** | **17.52** | **39** | **132** | **-2.67** | **169** | **0.008** |
| **PCS** | **30.03** | **35.59** | **8.42** | **9.34** | **37** | **132** | **-3.27** | **167** | **0.001** |
| MCS | 42.27 | 44.35 | 12.30 | 12.96 | 37 | 132 | -0.87 | 167 | 0.385 |
| **PF** | **22.97** | **44.89** | **26.60** | **31.00** | **37** | **132** | **-3.91** | **167** | **0.000** |
| RP | 30.07 | 39.20 | 29.53 | 30.38 | 37 | 132 | -1.63 | 167 | 0.106 |
| BP | 58.78 | 70.45 | 27.14 | 26.18 | 37 | 132 | -2.38 | 167 | 0.019 |
| **GH** | **20.81** | **29.39** | **22.31** | **23.65** | **37** | **132** | **-1.97** | **167** | **0.050** |
| VT | 13.51 | 18.37 | 20.91 | 22.25 | 37 | 132 | -1.19 | 167 | 0.236 |
| **SF** | **55.41** | **68.75** | **35.42** | **37.37** | **37** | **132** | **-1.94** | **167** | **0.054** |
| RE | 59.46 | 69.60 | 35.89 | 30.67 | 37 | 132 | -1.71 | 167 | 0.089 |
| MH | 55.07 | 58.71 | 20.72 | 24.27 | 37 | 132 | -0.83 | 167 | 0.407 |

BP: Body Pain, GH: General Health, MCS: Mental Health Composite Scale, MH: Mental Health, PCS: Physical Composite Scale, PF: Physical Functioning, RE: Role Emotional, RP: Role Physical, SF: Social Functioning, VT: Vitality

red colour values: *p* < .05

blue colour values: *p* < .01

Paresis and quality of life domains

| Dependent variables | Mean | | Standard Deviation | | N | | t | df | p |
| --- | --- | --- | --- | --- | --- | --- | --- | --- | --- |
|  | yes | no | yes | no | yes | no |  |  |  |
| **EQ mobility** | **1.92** | **1.54** | **0.61** | **0.53** | **95** | **76** | **4.24** | **169** | **0.000** |
| **EQ self-care** | **1.96** | **1.24** | **0.77** | **0.46** | **95** | **76** | **7.20** | **169** | **0.000** |
| **EQ usual activities** | **2.18** | **1.49** | **0.74** | **0.60** | **95** | **76** | **6.58** | **169** | **0.000** |
| **EQ pain/discomfort** | **1.84** | **1.63** | **0.47** | **0.63** | **95** | **76** | **2.51** | **169** | **0.013** |
| EQ anxiety/depression | 1.84 | 1.72 | 0.57 | 0.64 | 95 | 76 | 1.27 | 169 | 0.205 |
| EQ VAS | 51.56 | 56.84 | 19.71 | 17.51 | 95 | 76 | -1.83 | 169 | 0.069 |
| **PCS** | **30.85** | **38.68** | **8.17** | **9.06** | **93** | **76** | **-5.90** | **167** | **0.000** |
| MCS | 43.92 | 43.87 | 12.37 | 13.41 | 93 | 76 | 0.02 | 167 | 0.980 |
| **PF** | **27.42** | **55.59** | **27.35** | **29.04** | **93** | **76** | **-6.48** | **167** | **0.000** |
| **RP** | **26.88** | **49.84** | **27.52** | **28.98** | **93** | **76** | **-5.27** | **167** | **0.000** |
| BP | 64.78 | 71.71 | 27.89 | 24.95 | 93 | 76 | -1.68 | 167 | 0.094 |
| GH | 24.62 | 31.05 | 23.60 | 23.18 | 93 | 76 | -1.78 | 167 | 0.078 |
| **VT** | **13.98** | **21.38** | **18.60** | **25.07** | **93** | **76** | **-2.20** | **167** | **0.029** |
| SF | 62.37 | 70.07 | 39.64 | 33.92 | 93 | 76 | -1.34 | 167 | 0.182 |
| RE | 63.71 | 71.87 | 34.09 | 28.95 | 93 | 76 | -1.66 | 167 | 0.100 |
| MH | 57.12 | 58.88 | 22.83 | 24.48 | 93 | 76 | -0.48 | 167 | 0.630 |

BP: Body Pain, GH: General Health, MCS: Mental Health Composite Scale, MH: Mental Health, PCS: Physical Composite Scale, PF: Physical Functioning, RE: Role Emotional, RP: Role Physical, SF: Social Functioning, VT: Vitality

red colour values: *p* < .05

blue colour values: *p* < .01

Anxiety and quality of life domains

| Dependent variables | Mean | | Standard Deviation | | N | | t | df | p |
| --- | --- | --- | --- | --- | --- | --- | --- | --- | --- |
|  | 0-7 | 11-21 | 0-7 | 11-21 | 0-7 | 11-21 |  |  |  |
| **EQ mobility** | **1.61** | **1.82** | **0.54** | **0.58** | **80** | **55** | **-2.11** | **133** | **0.037** |
| EQ self-care | 1.52 | 1.69 | 0.66 | 0.79 | 80 | 55 | -1.33 | 133 | 0.187 |
| EQ usual activities | 1.76 | 2.00 | 0.72 | 0.79 | 80 | 55 | -1.81 | 133 | 0.072 |
| **EQ pain/discomfort** | **1.61** | **1.89** | **0.56** | **0.53** | **80** | **55** | **-2.89** | **133** | **0.005** |
| **EQ anxiety/depression** | **1.59** | **2.09** | **0.57** | **0.62** | **80** | **55** | **-4.89** | **133** | **0.000** |
| **EQ VAS** | **60.73** | **49.18** | **16.54** | **17.47** | **80** | **55** | **3.89** | **133** | **0.000** |
| PCS | 35.08 | 33.52 | 9.54 | 8.55 | 80 | 55 | 0.97 | 133 | 0.335 |
| **MCS** | **51.40** | **33.65** | **9.85** | **10.35** | **80** | **55** | **10.08** | **133** | **0.000** |
| **PF** | **45.31** | **33.18** | **30.32** | **30.07** | **80** | **55** | **2.29** | **133** | **0.023** |
| RP | 39.38 | 31.59 | 31.89 | 28.04 | 80 | 55 | 1.46 | 133 | 0.146 |
| **BP** | **78.44** | **56.36** | **23.11** | **26.88** | **80** | **55** | **5.10** | **133** | **0.000** |
| **GH** | **37.31** | **15.18** | **22.18** | **19.63** | **80** | **55** | **5.97** | **133** | **0.000** |
| **VT** | **25.94** | **7.27** | **24.66** | **13.33** | **80** | **55** | **5.12** | **133** | **0.000** |
| **SF** | **78.75** | **45.00** | **33.78** | **35.16** | **80** | **55** | **5.61** | **133** | **0.000** |
| **RE** | **81.72** | **47.50** | **24.76** | **29.40** | **80** | **55** | **7.31** | **133** | **0.000** |
| **MH** | **72.66** | **38.86** | **18.67** | **17.46** | **80** | **55** | **10.61** | **133** | **0.000** |

BP: Body Pain, GH: General Health, MCS: Mental Health Composite Scale, MH: Mental Health, PCS: Physical Composite Scale, PF: Physical Functioning, RE: Role Emotional, RP: Role Physical, SF: Social Functioning, VT: Vitality

red colour values: *p* < .05

blue colour values: *p* < .01

Depression and quality of life domains

| Dependent variables | Mean | | Standard Deviation | | N | | t | df | p |
| --- | --- | --- | --- | --- | --- | --- | --- | --- | --- |
|  | 0-7 | 11-21 | 0-7 | 11-21 | 0-7 | 11-21 |  |  |  |
| **EQ mobility** | **1.36** | **2.07** | **0.48** | **0.53** | **69** | **67** | **-8.18** | **134** | **0.000** |
| **EQ self-care** | **1.22** | **2.13** | **0.45** | **0.69** | **69** | **67** | **-9.17** | **134** | **0.000** |
| **EQ usual activities** | **1.39** | **2.39** | **0.52** | **0.67** | **69** | **67** | **-9.68** | **134** | **0.000** |
| **EQ pain/discomfort** | **1.57** | **1.91** | **0.53** | **0.51** | **69** | **67** | **-3.86** | **134** | **0.000** |
| **EQ anxiety/depression** | **1.52** | **2.03** | **0.56** | **0.58** | **69** | **67** | **-5.22** | **134** | **0.000** |
| **EQ VAS** | **63.23** | **44.10** | **15.65** | **17.28** | **69** | **67** | **6.77** | **134** | **0.000** |
| **PCS** | **38.99** | **29.16** | **8.84** | **8.11** | **69** | **67** | **6.76** | **134** | **0.000** |
| **MCS** | **50.34** | **37.02** | **10.56** | **11.14** | **69** | **67** | **7.16** | **134** | **0.000** |
| **PF** | **57.61** | **20.52** | **26.54** | **26.45** | **69** | **67** | **8.16** | **134** | **0.000** |
| **RP** | **53.26** | **20.15** | **29.75** | **23.83** | **69** | **67** | **7.15** | **134** | **0.000** |
| **BP** | **77.54** | **56.72** | **23.15** | **26.68** | **69** | **67** | **4.87** | **134** | **0.000** |
| **GH** | **40.43** | **12.46** | **20.41** | **18.70** | **69** | **67** | **8.33** | **134** | **0.000** |
| **VT** | **28.99** | **5.97** | **24.49** | **13.12** | **69** | **67** | **6.80** | **134** | **0.000** |
| **SF** | **82.61** | **47.39** | **29.18** | **37.22** | **69** | **67** | **6.15** | **134** | **0.000** |
| **RE** | **83.70** | **47.95** | **25.30** | **30.20** | **69** | **67** | **7.49** | **134** | **0.000** |
| **MH** | **70.29** | **45.71** | **19.89** | **20.93** | **69** | **67** | **7.02** | **134** | **0.000** |

BP: Body Pain, GH: General Health, MCS: Mental Health Composite Scale, MH: Mental Health, PCS: Physical Composite Scale, PF: Physical Functioning, RE: Role Emotional, RP: Role Physical, SF: Social Functioning, VT: Vitality

red colour values: *p* < .05

blue colour values: *p* < .01

**Pearson r correlations between numeric variables and quality of life domains**

BP: Body Pain, GH: General Health, MCS: Mental Health Composite Scale, MH: Mental Health, PCS: Physical Composite Scale, PF: Physical Functioning, RE: Role Emotional, RP: Role Physical, SF: Social Functioning, VT: Vitality

|  | Age | Barthel Scale | MMSE |
| --- | --- | --- | --- |
| EQ mobility | ,21** | -.69** | -.44** |
| EQ self-care | .20** | -.79** | -.52** |
| EQ usual activities | .24** | -.69** | -.51** |
| EQ pain/discomfort | .17* | -.25** | -.23** |
| EQ anxiety/depression | -.02 | -.18* | -.18* |
| EQ VAS | -.06 | .33** | .31** |
| PCS | -.25** | .53** | .30** |
| MCS | .11 | .17* | .18* |
| PF | -.27** | .59** | .33** |
| RP | -.17* | .54** | .32** |
| BP | -.04 | .20** | .19* |
| GH | -.07 | .33** | .22** |
| VT | -.07 | .27** | .23** |
| SF | .08 | .22** | .14 |
| RE | -.09 | .43** | .33** |
| MH | .15* | .10 | .14 |

*: *p* < .05

**: *p* < .01

BP: Body Pain, GH: General Health, MCS: Mental Health Composite Scale, MH: Mental Health, PCS: Physical Composite Scale, PF: Physical Functioning, RE: Role Emotional, RP: Role Physical, SF: Social Functioning, VT: Vitality, MMSE: Mini Mental State Examination

**Multiple regression analysis**

**Results for the EQ-5D domains:**

Depended variable: EQ: mobility

|  | B | Beta | t | p |
| --- | --- | --- | --- | --- |
| (Constant) | 2.22 |  | 12.04 | <.001 |
| Barthel scale | -.01 | -.48 | -7.76 | <.001 |
| Limited daily activity before stroke | .32 | .22 | 4.03 | <.001 |
| HADS: depression | .03 | .25 | 3.99 | <.001 |

*F*(3, 165) = 71.80; *p* < *.*001

*R* = .75

*R^2^* = .57

HADS: Hospital Anxiety and Depression Scale

Depended variable: EQ: self-care

|  | B | Beta | t | p |
| --- | --- | --- | --- | --- |
| (Constant) | 2.69 |  | 13.48 | <.001 |
| Barthel scale | -.02 | -.60 | -11.30 | <.001 |
| Paresis | .23 | .16 | 3.15 | .002 |
| HADS: depression | .03 | .17 | 3.21 | .002 |
| Limited daily activity before stroke | .20 | .11 | 2.27 | .024 |

*F*(4, 164) = 93.30; *p* < *.*001

*R* = .83

*R^2^* = .69

HADS: Hospital Anxiety and Depression Scale

Depended variable: EQ usual activities

|  | B | Beta | t | p |
| --- | --- | --- | --- | --- |
| (Constant) | 2.59 |  | 9.53 | <.001 |
| Barthel Scale | -.01 | -.39 | -6.07 | <.001 |
| Limited daily activity before stroke | .41 | .23 | 4.13 | <.001 |
| HADS: depression | .04 | .23 | 3.88 | <.001 |
| MMSE: anxiety | -.02 | -.12 | -2.18 | .031 |
| Paresis | .18 | .12 | 2.14 | .034 |

*F*(5, 163) = 55.55; *p* < *.*001

*R* = .79

*R^2^* = .63

HADS: Hospital Anxiety and Depression Scale

MMSE: Mini Mental State Examination

Depended variable: EQ: pain/depression

|  | B | Beta | t | p |
| --- | --- | --- | --- | --- |
| (Constant) | .62 |  | 2.16 | .032 |
| Limited daily activity before stroke | .41 | .31 | 4.34 | <.001 |
| HADS: anxiety | .03 | .24 | 3.32 | .001 |
| Age | .01 | .15 | 2.09 | .038 |

*F*(3, 165) = 12.41; *p* < *.*001

*R* = .43

*R^2^* = .18

HADS: Hospital Anxiety and Depression Scale

Depended variable: EQ: anxiety/depression

|  | B | Beta | t | p |
| --- | --- | --- | --- | --- |
| (Constant) | 1.13 |  | 11.26 | <.001 |
| HADS: anxiety | .04 | .30 | 3.98 | <.001 |
| HADS: depression | .03 | .21 | 2.67 | .008 |
| Speech disorder | .27 | .18 | 2.65 | .009 |

*F*(3, 165) = 18.04; *p* < *.*001

*R* = .50

*R^2^* = .25

HADS: Hospital Anxiety and Depression Scale

Depended variable: EQ: VAS

|  | B | Beta | t | p |
| --- | --- | --- | --- | --- |
| (Constant) | 72.91 |  | 23.87 | <.001 |
| HADS: depression | -1.08 | -.29 | -3.66 | <.001 |
| HADS: anxiety | -.98 | -.22 | -2.87 | .005 |
| Subsequent stroke | -10.15 | -.16 | -2.27 | .025 |

*F*(3, 165) = 16.86; *p* < *.*001

*R* = .48

*R^2^* = .23

HADS: Hospital Anxiety and Depression Scale

**Results for the SF-12 survey domains:**

BP: Body Pain, GH: General Health, MCS: Mental Health Composite Scale, MH: Mental Health, PCS: Physical Composite Scale, PF: Physical Functioning, RE: Role Emotional, RP: Role Physical, SF: Social Functioning, VT: Vitality

Depended variable: PCS

|  | B | Beta | t | p |
| --- | --- | --- | --- | --- |
| (Constant) | 33.37 |  | 9.46 | <.001 |
| Barthel Scale | .11 | .28 | 3.87 | <.001 |
| Limited daily activity before stroke | -3.14 | -.14 | -2.04 | .043 |
| HADS: depression | -.44 | -.23 | -3.19 | .002 |
| Paresis | -3.29 | -.17 | -2.55 | .012 |
| Previous stroke | -5.02 | -.14 | -2.28 | .024 |

*F*(5, 163) = 24.25; *p* < *.*001

*R* = .65

*R^2^* = .43

HADS: Hospital Anxiety and Depression Scale

Depended variable: MCS

|  | B | Beta | t | p |
| --- | --- | --- | --- | --- |
| (Constant) | 64.41 |  | 38.70 | <.001 |
| HADS: anxiety | -1.62 | -.53 | -8.70 | <.001 |
| HADS: depression | -.80 | -.31 | -5.04 | <.001 |

*F*(2, 166) = 91.81; *p* < *.*001

*R* = .72

*R^2^* = .53

HADS: Hospital Anxiety and Depression Scale

Depended variable: PF

|  | B | Beta | t | p |
| --- | --- | --- | --- | --- |
| (Constant) | 37.42 |  | 3.33 | .001 |
| Barthel Scale | .38 | .29 | 3.98 | <.001 |
| HADS: depression | -2.12 | -.33 | -5.10 | <.001 |
| Paresis | -15.31 | -.24 | -4.07 | <.001 |
| Speech disorder | -8.80 | -.12 | -1.98 | .049 |

*F*(4, 164) = 41;14 *p* < *.*001

*R* = .71

*R^2^* = .50

HADS: Hospital Anxiety and Depression Scale

Depended variable: RP

|  | B | Beta | t | p |
| --- | --- | --- | --- | --- |
| (Constant) | 27.91 |  | 2.53 | .012 |
| Barthel Scale | .43 | .33 | 4.63 | <.001 |
| HADS: depression | -1.77 | -.29 | -3.95 | <.001 |
| Limited daily activity before stroke | -13.88 | -.19 | -2.97 | .003 |

*F*(3, 165) = 37;94 *p* < *.*001

*R* = .64

*R^2^* = .41

HADS: Hospital Anxiety and Depression Scale

Depended variable: BP

|  | B | Beta | t | p |
| --- | --- | --- | --- | --- |
| (Constant) | 89.18 |  | 16.31 | <.001 |
| HADS: depression | -1.50 | -.28 | -3.59 | <.001 |
| HADS: anxiety | -1.75 | -.27 | -3.53 | .001 |
| Education | 4.41 | .14 | 2.00 | .047 |

*F*(3, 165) = ; *p* < *.*001

*R* = .49

*R^2^* = .24

HADS: Hospital Anxiety and Depression Scale

Depended variable: GH

|  | B | Beta | t | p |
| --- | --- | --- | --- | --- |
| (Constant) | 59.48 |  | 17.21 | <.001 |
| HADS: depression | -1.93 | -.40 | -5.84 | <.001 |
| HADS: anxiety | -1.69 | -.30 | -4.37 | <.001 |
| Previous stroke | -11.96 | -.13 | -2.14 | .034 |

*F*(3, 165) = 36;67 *p* < *.*001

*R* = .63

*R^2^* = .40

HADS: Hospital Anxiety and Depression Scale

Depended variable: VT

|  | B | Beta | t | p |
| --- | --- | --- | --- | --- |
| (Constant) | 48.94 |  | 12.54 | <.001 |
| HADS: depression | -1.87 | -.42 | -5.52 | <.001 |
| Limited daily activity before stroke | -10.36 | -.20 | -2.95 | .004 |
| HADS: anxiety | -.86 | -.16 | -2.28 | .024 |

*F*(3, 165) = 31;64 *p* < *.*001

*R* = .60

*R^2^* = .37

HADS: Hospital Anxiety and Depression Scale

Depended variable: SF

|  | B | Beta | t | p |
| --- | --- | --- | --- | --- |
| (Constant) | 111.58 |  | 19.17 | <.001 |
| HADS: depression | -2.69 | -.35 | -4.86 | <.001 |
| HADS: anxiety | -2.65 | -.30 | -4.07 | <.001 |

*F*(2, 166) = 37.92; *p* < *.*001

*R* = .56

*R^2^* = .31

HADS: Hospital Anxiety and Depression Scale

Depended variable: RE

|  | B | Beta | t | p |
| --- | --- | --- | --- | --- |
| (Constant) | 76.14 |  | 7.16 | <.001 |
| HADS: depression | -1.62 | -.25 | -3.14 | .002 |
| HADS: anxiety | -2.86 | -.37 | -5.52 | <.001 |
| Barthel Scale | .35 | .26 | 3.68 | <.001 |

*F*(3, 165) = 43;65 *p* < *.*001

*R* = .67

*R^2^* = .44

HADS: Hospital Anxiety and Depression Scale

Depended variable: MH

|  | B | Beta | t | p |
| --- | --- | --- | --- | --- |
| (Constant) | 112.11 |  | 16.15 | <.001 |
| HADS: anxiety | -2.91 | -.52 | -8.61 | <.001 |
| HADS: depression | -1.89 | -.40 | -5.61 | <.001 |
| Barthel Scale | -.16 | -.16 | -2.54 | .012 |

*F*(3, 165) = ; *p* < *.*001

*R* = .75

*R^2^* = .56

HADS: Hospital Anxiety and Depression Scale

**Supplementary Material 2**

**Figure 1. Summary of the SF-12 survey results**


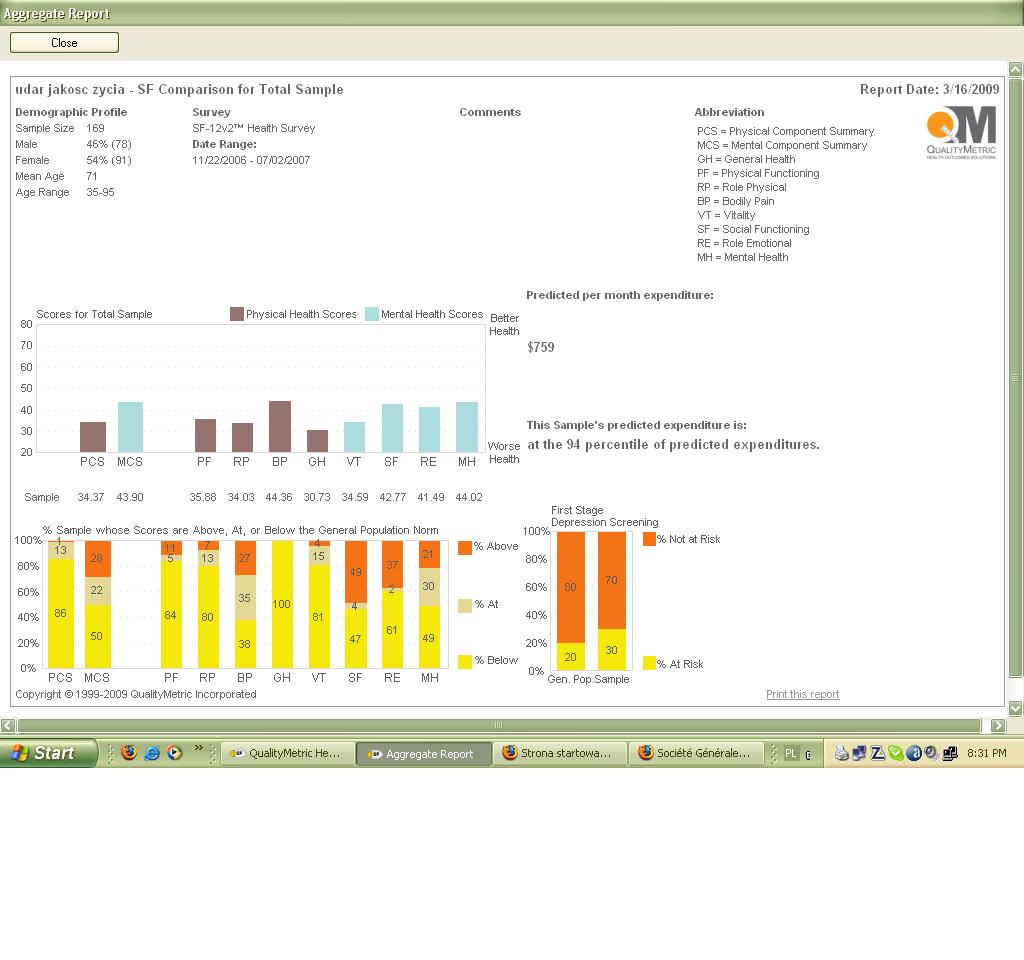


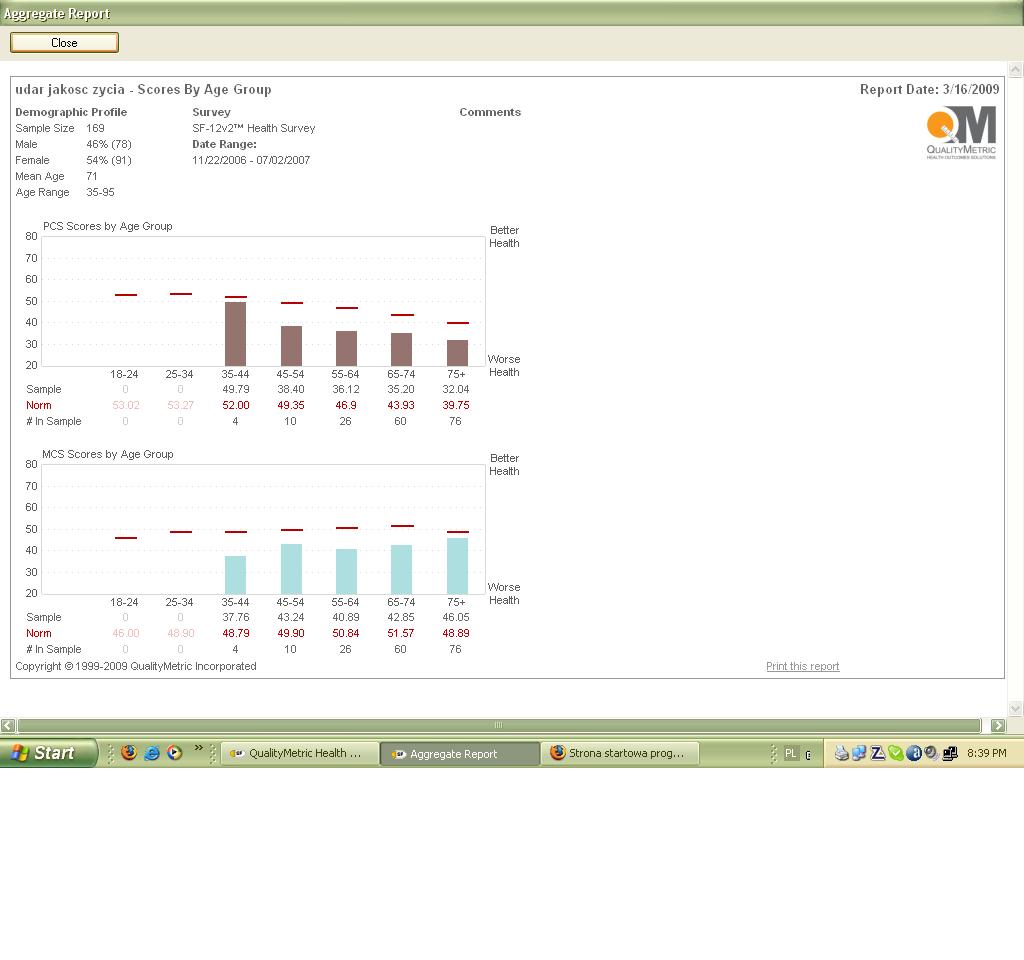
**Figure 2. SF-12 result by patients’ age**

**Figure 3. SF-12 result by patients’ gender**


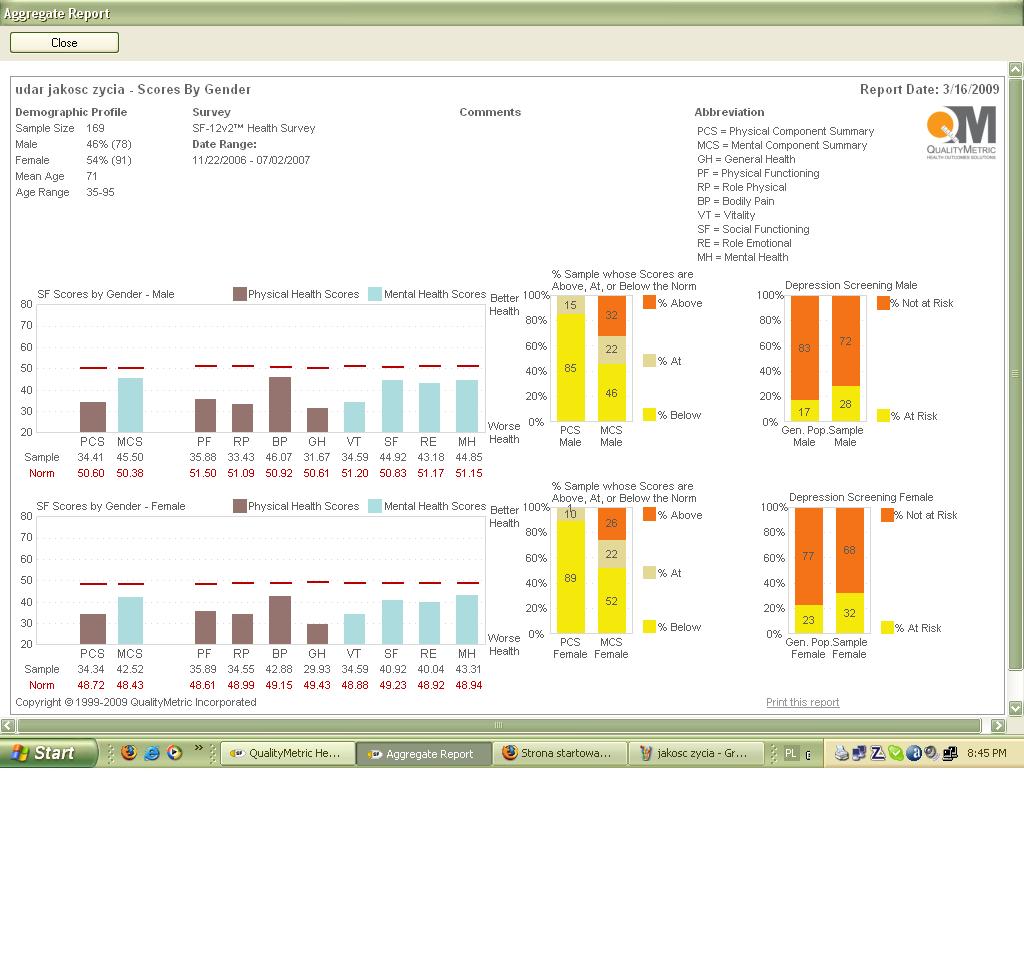

Supplement: Supplemental Material [file ZJMA_A_1775933_SM8012.docx]
